# The interaction between cellular senescence and chronic kidney disease as a therapeutic opportunity

**DOI:** 10.3389/fphar.2022.974361

**Published:** 2022-08-26

**Authors:** Jing-Li Zhao, Xiao-Hui Qiao, Jian-Hua Mao, Fei Liu, Hai-Dong Fu

**Affiliations:** ^1^ Department of Nephrology, The Children’s Hospital, Zhejiang University School of Medicine, National Clinical Research Center for Child Health, Hangzhou, China; ^2^ Department of Pediatric Internal Medicine, Ningbo Women and Children’s Hospital, Ningbo, China

**Keywords:** chronic kidney disease, cellular senescence, interaction, mechanism, therapy

## Abstract

Chronic kidney disease (CKD) is an increasingly serious public health problem in the world, but the effective therapeutic approach is quite limited at present. Cellular senescence is characterized by the irreversible cell cycle arrest, senescence-associated secretory phenotype (SASP) and senescent cell anti-apoptotic pathways (SCAPs). Renal senescence shares many similarities with CKD, including etiology, mechanism, pathological change, phenotype and outcome, however, it is difficult to judge whether renal senescence is a trigger or a consequence of CKD, since there is a complex correlation between them. A variety of cellular signaling mechanisms are involved in their interactive association, which provides new potential targets for the intervention of CKD, and then extends the researches on senotherapy. Our review summarizes the common features of renal senescence and CKD, the interaction between them, the strategies of senotherapy, and the open questions for future research.

## 1 Introduction

Chronic kidney disease (CKD) is a group of chronic diseases caused by inflammation, metabolic disorders, toxins and other various factors (Boor et al., 2010). It afflicts more than 13% of the world’s population (Hill et al., 2016). It is generally characterized by progressive glomerulosclerosis, tubular atrophy, interstitial fibrosis and renal failure, as well as non-renal complications (Dai et al., 2019). What could be the root cause(s) of the persistence of renal injury, multi-organ involvement and the final renal failure in CKD? Among multiple explanations, the effect of cellular senescence on CKD has been gaining attention (Xu et al., 2020).

Cellular senescence is defined as the permanent cessation of cell proliferation (Kuilman et al., 2010) and is used to describe ageing on cellular level. It is characterized by the stable cell cycle arrest, apoptosis inhibition, sustained high metabolic rate and a pro-inflammatory state called senescence associated secretory phenotype (SASP) (Di Micco et al., 2021). Actually, kidney is one of the most significantly affacted organs during the process of natural ageing (Long et al., 2005). Renal ageing and senescence lead to renal pathophysiological changes and systemic geriatric phenotypes, which are similar to those of CKD. It should be noted that renal senescence can also occur in sick children and may reduce their renal regeneration potential (Melk et al., 2009).

In view of the similarities between CKD and renal senescence, it is speculated that they are closely related (Kubben and Misteli, 2017). In fact, senescence is strongly associated not only with the development of CKD, but also with the progression of CKD, and vice versa (Schroth et al., 2020). Though effective treatments to halt or reverse CKD are extremely limited, regulating renal senescence is expected to provide a new target for its intervention.

In present review, the evidences for the interactive association of renal senescence and CKD, potential mechanisms that might explain this association, therapeutic approaches targeting senescence for the intervention of CKD, and the prospects for the future are discussed.

## 2 Basic concepts of cellular senescence

**FIGURE 1:**
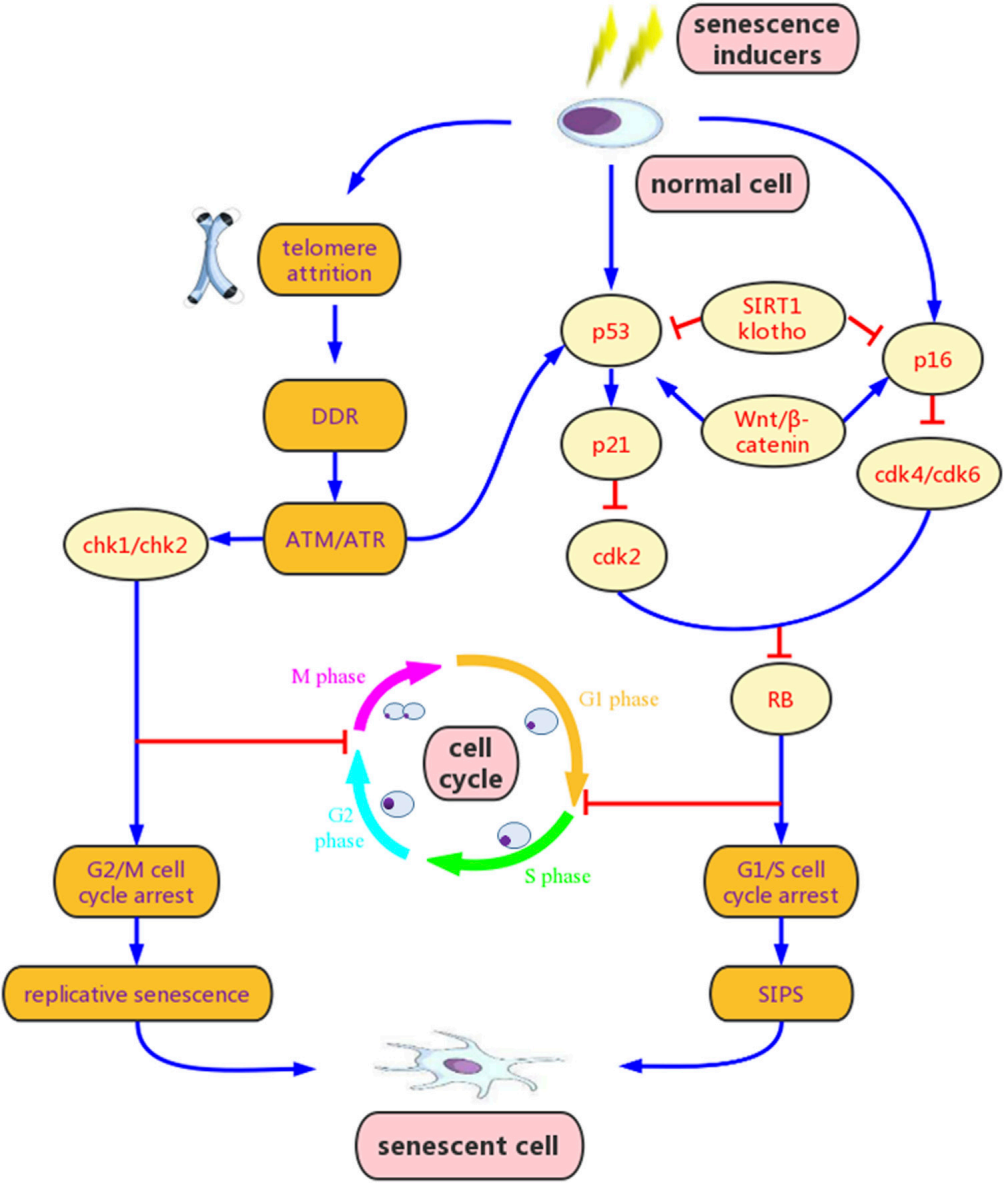
Mechanisms of cellular senescence. Senescence inducers, such as oxidative stress, DNA damage, mitochondrial dysfunction and epigenetic stress, can activate the ataxia telangiectasia mutated/ataxia telangiectasia and Rad3-related (ATM/ATR) signaling and other multiple pathways, resulting in p53 phosphorylation and increased p21 transcription, and/or p16 over-experession. Activation of p21 and p16 inhibits cyclin-dependent kinase 2 (CDK2) or CDK4/CDK6 and prevents retinoblastoma (RB) phosphorylation, leading to G1/S cell cycle arrest. Additionally, activated ATM/ATR signaling can also induce G2/M cell cycle arrest via checkpoint kinase 1 (Chk1) and Chk2. Wnt/β-catenin promotes senescence by stimulating p53 and p16, while klotho and sirtuins 1 (SIRT1) inhibit senescence by blocking these pathways. Cellular senescence initially leads to elevated senescence-associated β-galactosidase (SA-β-gal) and senescence associated secretory phenotype (SASP) release. However, if senescence persisists, it may evolve to chronic senescence and secondary senescence, and contributes to various degradations. DDR, DNA damage response; SIPS, stress-induced premature senescence.

Traditionally, cellular senescence has been divided into two types, namely Hayflick-type replicative senescence, characterized by the telomere attrition (Hayflick and Moorhead, 1961), and stress-induced premature senescence (SIPS), caused by various stimuli (von Zglinicki, 2002; Sedelnikova et al., 2004; Wiley et al., 2016; Petrova et al., 2016). A variety of markers have been used to identify cellular senescence, among which the senescence-associated β-galactosidase (SA-β-gal) activity at pH 6.0, cyclin-dependent kinase (CDK) inhibitors such as p16^ink4a^ (hereafter referred as p16) and p21^CIP1^ (hereafter referred as p21), and SASPs are the most common ones (Hernandez-Segura et al., 2018). The p53/p21 and p16/retinoblastoma (RB) pathways are the most critical signaling pathways that are related to cellular senescence (Calcinotto et al., 2019) (Figure 1). Another important factor leading to the expansion and spread of cellular senescence is SASPs, which are a series of pro-inflammatory and pro-fibrotic factors secreted by senescent cells (Valentijn et al., 2018), such as interleukin-1 (IL-1), IL-6, IL-8, transforming growth factors-β (TGF-β), plasminogen activator inhibitor (PAI), and insulin-like growth factor-1 (IGF-1). SASPs enable the primary senescent cells to direct adjacent or distant nonsenescent cells to experience secondary senescence in autocrine, paracrine, and juxtacrine manners (Admasu et al., 2021). Another function of the SASPs is to activate immune surveillance and recruit immune cells to eliminate senescent cells (Faget et al., 2019). However, the accumulation of senescent cells often gradually exceeds the clearance capacity of the immune cells, contributing to the development of senescence.

## 3 The association between renal senescence and CKD

As mentioned above, renal ageing and senescence share numerous similarities with CKD in renal and systemic manifestations. Besides, the similarities are also reflected in their pathogenic mechanisms, such as the secretion of pro-inflammatory and pro-fibrotic factors, oxidative stress, mitochondrial dysfunction, and loss of renoprotective factors (O’Sullivan et al., 2017). It is reported that SASP and CKD-associated secretory phenotype appear to have a lot in common (Wang et al., 2017). Renal fibrosis is regarded as the main determinant of the gradual loss of renal function and the prognosis of CKD (Higgins et al., 2018). The cytokine mediated signaling pathways, such as the TGF-β/Smad pathway and the Wnt pathway, which play important roles in renal fibrosis (Isaka 2018; Luo et al., 2018), are also involved in renal senescence. In addition, the immune deficiency in CKD is analogous to immunosenescence (Sato and Yanagita, 2019).

### 3.1 Evidence for renal senescence in CKD

The characteristics of cellular senescence are presented in all parts of renal parenchyma in CKD patients and animal models (Dai et al., 2019; Wang et al., 2021). P53 is over-expressed in the lymphocytes of CKD patients, and the mesenchymal stem cells from CKD rats are prematurely senescent (Klinkhammer et al., 2014). Because the renal functional reserve is gradually impaired, renal senescence undoubtedly increases the susceptibility to CKD (Nitta et al., 2013). Indeed, the average prevalence of CKD in the elderly is significantly higher than that in the young (Prakash and O’Hare, 2009). The fact that elevated p16 level and SA-β-gal activity often precede the renal changes in different stages of CKD and CKD-related renal diseases (Li and Wang, 2018), p21 level is significantly up-regulated in human transplanted kidney undergoing AKI-to-CKD transition (Cippà et al., 2018), the pathological changes of renal fibrosis, inflammation and microvascular rarefaction in the elderly mice are more significant than those in the young control group in the ischemia-reperfusion injury (IRI) model of CKD (Clements et al., 2013), and p21 knockout ameliorates progression to CKD in mouse models (Megyesi et al., 1999), suggesting that renal senescence is involved in the pathogenesis and progression of CKD.

### 3.2 Evidence for CKD in renal senescence

Compared with the general population, the renal ageing and senescence process is greatly accelerated and advanced in CKD patients (Dai et al., 2019). Besides, a high prevalence of senescent cells has been noticed in renal biopsies of young patients with diverse CKD (Halloran and Melk, 2001). Moreover, the shortening of telomeres and the increase of SA-β-gal levels in collecting tubules of CKD cats are more significant than those in general cats, whether young or aged (Quimby et al., 2013).

## 4 Potential mechanisms for association of renal senescence and CKD

Various animal models have been used to study the association between renal senescence and CKD or CKD-related renal diseases (Table 1). As mentioned above, renal senescence may be both the cause and the consequence of CKD. Acute senescence is a protective response to various renal insults, which plays a role in promoting immune clearance and tissue repair (van Deursen.., 2014). However, if senescent cells are not cleared in time, they will gradually accumulate, and may induce chronic senescence. As reported by various studies, senescent cell accumulation contributes to SASPs secretion and signaling, abnormal renal repair, and renal fibrosis (Braun et al., 2012; Liu et al., 2012; Cippà et al., 2018), further leading to CKD and its systemic complications (Schroth et al., 2020). In turn, various pathological products of CKD stimulate the kidney to remain in a state of chronic inflammation, oxidative stress and metabolic abnormality, which promotes the induction and accumulation of chronic senescent cells.

**TABLE 1:** Animal model studies on the relationship between cellular senecence and chronic kidney disease (CKD).

Model	Intervention	Effect of intervention	Indication
renal IRI Lee et al., (2012)	p16^ink4a^/p19^ARF^ double KO	improved epithelial repair, renal fibrosis and inflammation	Reduced senescence has a renoprotective effect in AKI
renal Tx Braun et al., (2012)	p16^ink4a^ KO	less atrophy and fibrosis after Tx	Inhibiting senescence have therapeutic benefit in kidney transplantation
DN Wolf et al., (2005)	p27^kip1^ KO	reduced glomerular hypertrophy and tubule-interstitial lesion	Inhibiting senescence by deletion of p27^Kip1^, an inhibitor of CDKs, attenuates the functional and morphologic features of DN.
DN Al-Douahji et al., (1999)	p21^cip1^ KO	mitigated proteinuria and glomerular expansion	Inhibiting senescence ameliorates glomerular hypertrophy in DN, which is protective of renal function
CKD Chang et al., (2016)	upregulate klotho	reduced vascular calcification	Inhibiting senescence by upregulating α-klotho attenuates vascular calcification in CKD.
CKD Hum et al., (2017)	stable delivery of AAV expressing klotho	reduced hyperphosphatemia	Inhibiting senescence by sustained klotho treatment reduces hyperphosphatemia in CKD.
CKD Hu et al., (2011)	transgenic overexpressing klotho	enhanced renal function and less calcification	Inhibiting senescence by overexpressing klotho ameliorates vascular calcification and preserves renal function in CKD.
chronic GN Haruna et al., (2007)	klotho transgene	reduced proteinuria and improved renal function	Inhibiting senescence by genetic manipulation of klotho gene ameliorates progressive renal injury in CKD.
post-AKI CKD Shi et al., (2016)	recombinant αklotho administration	accelerated renal recovery and reduced renal fibrosis	Inhibiting senescence by αklotho overexpression mitigates renal fibrosis and retards AKI progression to CKD.
UUO Gong et al., (2021)	knockdown of BRG-1	reduced renal fibrosis	Reduced senescence attenuates renal fibrosis in CKD.
UUO Adis et al., (2013)	rhEPO	mitigated tubular epithelial cell regeneration and renal fibrosis	Inhibiting senescence by erythropoietin preserves tubular epithelial cell regeneration and ameliorates renal fibrosis in CKD.
telomerase deficient Westhoff et al., (2010)	renal IRI	higher expression of p21, and reduced cellular regeneration	IRI leads to increased senescence

Multiple animal models have been used to study the association between CKD and renal senescence. Since CKD may be caused by various renal diseases, especially acute kidney injury (AKI), glomerulonephritis (GN) and diabetes nephropathy (DN), those CKD-related renal diseases are also included in the research on the association between CKD and renal senescence. This table summarizes several of these studies, and describes the models and the interventions that are used in them, as well as the effects of the interventions. AAV, adeno-associated virus; BRG-1, brahma-related gene-1; CDK, cyclin-dependent kinase; EMT, epithelial-to-mesenchymal transition; IRI, ischemia-reperfusion injury; KO, knock-out; rhEPO, recombinant huma erythropoietin; Tx, transplant; UUO, unilateral ureteric obstruction.

### 4.1 Renal senescence promoting CKD

**FIGURE 2:**
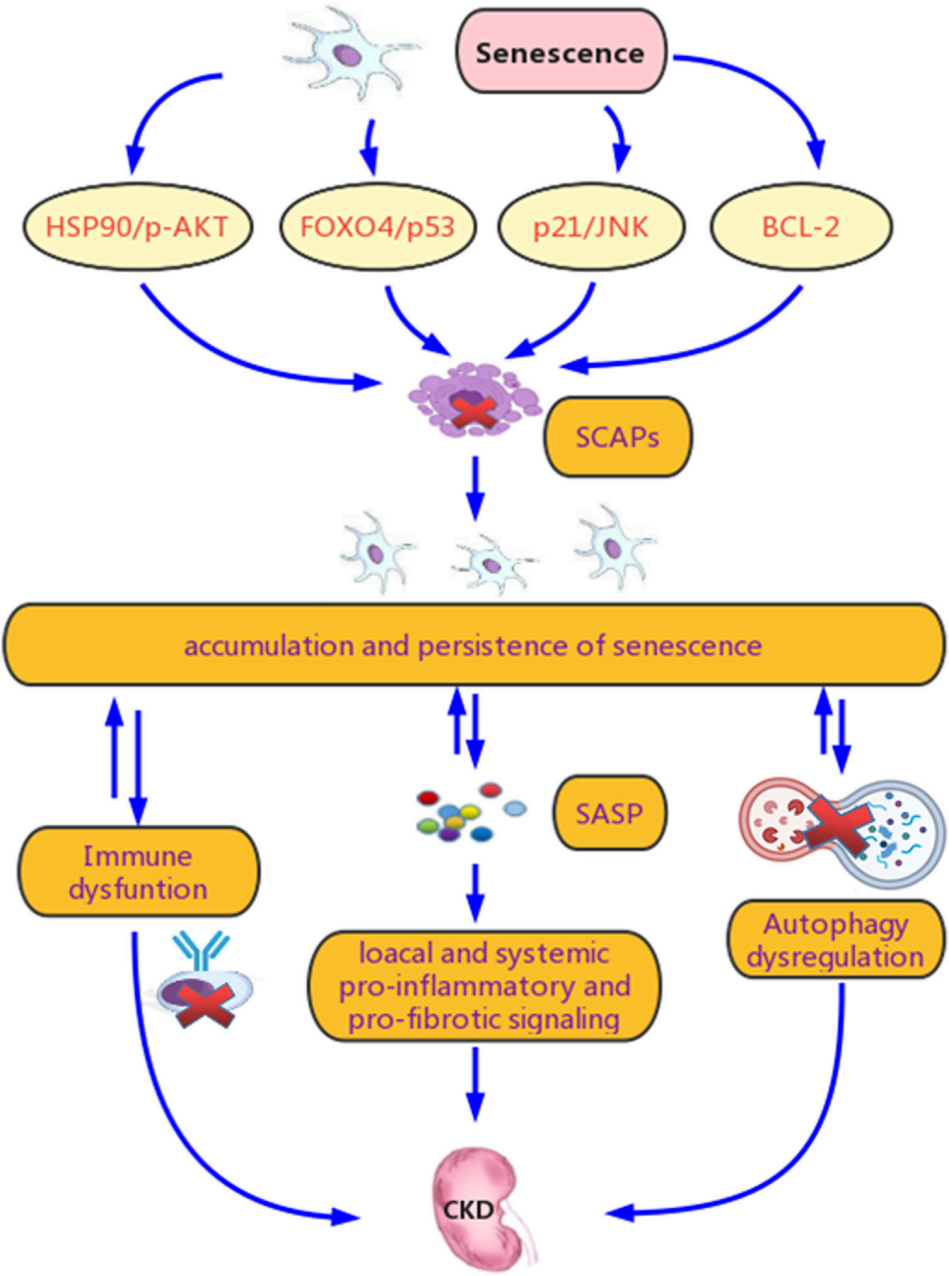
Potential mechanisms for senescence promoting chronic kidney disease (CKD). Renal senscence and CKD are tightly connected. Chronic stimulation of various stressors in CKD leads to the continuous and excessive induction of chronic senescent cells and relaease of senescence associated secretory phenotype (SASP), which contributes to their accumulation and persistence. Another crucial reason for their persistence is senescent cell anti-apoptotic pathways (SCAPs), which prevents senescent cells from clearance mainly though the B-cell lymphoma-2 (BCL-2), Forkhead box O4 (FOXO4)/p53, p21/JNK and HSP90/p-AKT pathways. Meanwhile, this persistence promotes SASP secretion and spread, induces abnormal renal repair, and exacerbate renal fibrosis, culminating in CKD progression and its systemic complications. Dysregulation of autophagy and immune system are involved in both the persistence of senescence and the progression of CKD.

The persistence of chronic senescent cells plays a critical role in the correlation between renal senescence and CKD (Figure 2). The interactions between cell cycle regulators and inhibitors, changes in the balance between pro-apoptotic and anti-apoptotic factors, and metabolic abnormalities may explain this persistence and its promoting effect on CKD.

#### 4.1.1 Senescent cell anti-apoptotic pathways

5SCAPs refer to mechanisms that contribute to the prolonged survival of senescent cells (Wang et al., 2021). Activation of the BCL-2 family, ephrin ligand B1 (EFNB1), EFNB3, Forkhead box O-4 (FOXO-4), HSP90/p-AKT, and p21/JNK plays an important role in SCAPs (Wang et al., 2021). BCL-2 inhibits autophagy by interacting with autophagy protein Beclin1 and suppressing the formation of autophagosome (Yosef et al., 2016; Goligorsky, 2020). FOXO-4 is a p53 sequester in the nucleus, which can restrict p53-mediated apoptosis (Baar et al., 2017). The activation of p21 prevents senescent cells from apoptosis by limiting JNK signaling and caspase (Yosef et al., 2017). Stabilized p-AKT by HSP90 also contributes to the prolonged survival of senescent cells (Fuhrmann-Stroissnigg et al., 2017). In fact, agents targeting the SCAPs can inhibit renal senescence and decline of renal function in chronological and transgenic ageing mice (Baar et al., 2017), indicating a relationship between SCAPs and CKD.

#### 4.1.2 Autophagy dysregulation

Autophagy is a highly conserved process of cellular degradation and recycling. Thus, impaired autophagy will lead to the persistence and accumulation of senescent cells. The role of autophagy in cellular senescence is related to αklotho (Shi et al., 2016), telomerase (Harris and Cheng, 2016) and adenosine monophosphate-activated protein kinase (AMPK)/rapamycin (mTOR) pathway (Goligorsky, 2020). Autophagy dysregulation is also involved in promoting CKD (Lenoir et al., 2016). It has been shown that knockout of rubicon, a negative regulator of autophagy (Nakamura et al., 2019), and the autophagic flux induced by calorie restriction (CR) (Schmitt and Melk, 2017) can slow down the process of renal tubular atrophy and interstitial fibrosis.

#### 4.1.3 SASPs

Continuous stimulation of SASPs is crucial in promoting CKD, because it contributes to the enhancement and propagation of senescent phenotypes, induces abnormal renal repair, and causes a decline in renal function. Besides, factors related to chronic release of SASPs are also known as pro-fibrotic factors (Goligorsky, 2020), such as TGF-β and Wnt. Therefore, SASPs are able to exacerbate renal fibrosis, and further lead to CKD deterioration (Goligorsky, 2020). In addition to their local effects on the kidney, SASPs also induce systemic transmission of pro-inflammatory and pro-fibrotic signaling, resulting in systemic phenotypes of CKD (Goligorsky, 2020).

#### 4.1.4 Immune system alterations

Abnormal activation of the innate immune system in CKD patients leads to increased pro-inflammatory cytokines (Sato and Yanagita, 2019) and senescence of renal tubular cells (Jin et al., 2017). The adaptive immune response of CKD patients is also affected, which is characterized by the increase of CD4^+^CD28^−^ cells (Lisowska et al., 2012), the decrease of regulatory T (Treg) cells (Lisowska et al., 2012) and immature B cells (Kim et al., 2012), a shift toward the pro-inflammatory Th1 differentiation (Litjens et al., 2006), and the decline of CD4/CD8 T cell ratio (Yoon et al., 2006). The accumulation of senescent cells is not only due to immune dysfunction, but also related to immune evasion (Pereira et al., 2019). In addition, infiltration of pro-inflammatory B cells and T cells conduce to a pro-fibrotic milieu, and induce renal fibrosis, leading to CKD progression (Lee et al., 2017).

### 4.2 CKD promoting the renal senescence

**FIGURE 3:**
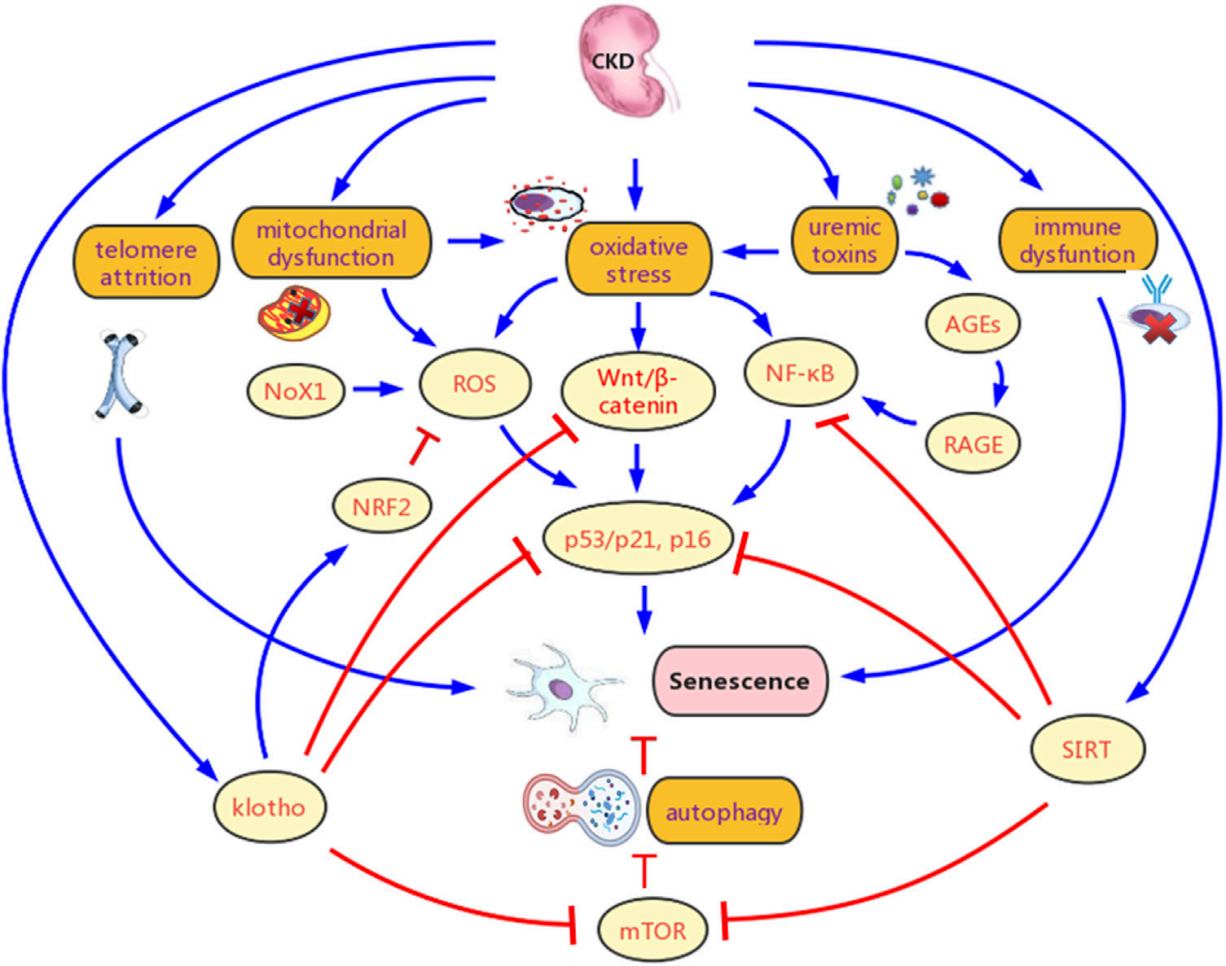
Potential mechanisms for chronic kidney disease (CKD) promoting senescence. CKD contributes to senescence mainly by Multiple pathological products of CKD leads to chronic inflammation, oxidative stress, and metabolic abnormality in the kidney, which contributes to senescence. Besides, telomere attrition, klotho defect, sirtuins (SIRTs) deficiency, autophagy inhibition, and immune dysfunction are also important causes for increased senescence. Various signaling pathways are involved in the promotion of senescence in CKD, mainly include factor-erythroid 2-related factor 2 (NRF2), Wnt/β-catenin, NF-κB, and mTOR. AGEs, advanced glycation end products; RAGE, AGE-receptor for advanced glycation end products; ROS, reactive oxygen species.

The potential mechanisms for CKD promoting renal senescence are showed in Figure 3, and the details are as follows.

#### 4.2.1 Telomere attrition

Stresses induced by multiple kidney injuries in CKD accelerate telomere attrition, subsequently leading to increased replicative senescence (Wills and Schnellmann, 2011). Studies have shown that the telomere lengths of T cells in patients with end stage renal disease are shorter than those in healthy group (Hirashio et al., 2014), the telomerase activities of peripheral blood mononuclear cells (PBMC) increase with the progression of CKD (Kidir et al., 2017), and Poly (A)-specific ribonuclease (PARN) mutation, a key cause of telomere abnormality-related diseases, is prevalent in CKD patients (Lata et al., 2018), which indicating that CKD is closely related to telomere dysfunction.

#### 4.2.2 Oxidative stress and inflammatory burden

Systemic oxidative stress and inflammatory burden, caused by over-activation of renin-angiotension-aldosterone system (RAAS), reduction of antioxidant factors, hyperphosphatemia, or other various factors, is prevalent in CKD, which has been considered to be one of the key mechanisms leading to renal senescence (Qaisar et al., 2018). It is reported that the burden of inflammation in CKD children seems to be much higher than that in general children (Lambert et al., 2004). Increased metabolic rate and ATP consumption trigger mitochondrial dysfunction, consequently leading to reactive oxygen species (ROS) over-production (Tamaki et al., 2014), which is the basis of increased oxidative stress, even in the early stage of CKD. The nuclear factor-erythroid 2-related factor 2 (NRF2) is a key regulator of antioxidant enzymes. Senescence markers, such as p16, p21 and SASPs, are increased in NRF2-deficient mice (Fulop et al., 2018). In the PBMC of CKD, the decreased expression of NRF2 is accompanied by mitochondrial dysfunction (Liu et al., 2019) and up-regulation of pro-inflammatory factors, such as NF-κB (Stockler-Pinto et al., 2018). On the contrary, the NRF2 agonist bardoxolone inhibits senescence in the CKD mouse model (Nagasu et al., 2019).

#### 4.2.3 Uremic toxins

Uremic toxins, such as advanced glycation end products (AGEs), are accumulated in CKD due to increased generation and decreased clearance (Stinghen et al., 2016). Children with CKD also shows high circulating levels of AGEs (Misselwitz et al., 2002). AGE/AGE-receptor for advanced glycation end products (RAGE) axis activates the NF-κB pathway (Sanajou et al., 2018), induces endoplasmatic reticulum stress (Liu et al., 2014), inhibits autophagy (Shi et al., 2019), and promotes p16 (Liu et al., 2015) and p21 (Liu et al., 2014) expression, resulting in increased renal senescence. On the contrary, reduced senescence is observed in rodents over-expressing the AGE-detoxifying enzyme Glo-1 (Hirakawa et al., 2017).

#### 4.2.4 Klotho defect

Klotho, an anti-senescence single-pass transmembrane protein, is primarily expressed in the proximal and distal tubules of kidney (Zou et al., 2018). Klotho regulates cellular senescence mainly by attenuating p53/p21 and Wnt/β-catenin pathways (Kuro-o., 2019). In addition, klotho is involved in regulating the activity of many other pathways, such as TGF-β, NRF2, FGF23, and IGF-1 (Sopjani et al., 2015), thereby inhibiting cellular senescence. Studies have shown that klotho begins to decline in very early stages of CKD (Wang et al., 2021), and the TGF-β signaling plays a crucial role in down-regulating klotho in CKD (Zhou et al., 2013). Klotho defect leads to the increased cellular senescence and secretion of SASPs (Castilho et al., 2009), aggravates renal fibrosis and promotes a variety of systemic phenotypes.

#### 4.2.5 Sirtuins deficiency

Sirtuins (SIRTs) are a group of NAD^+^-dependent deacetylases (Morigi et al., 2018), which has a deep impact on a variety of cytokines and signaling pathways related to cellular senescence, such as FOXO, p53 (Li et al., 2019), NF-κB, NRF2/ARE pathway (Zhuang et al., 2021), PTEN-induced putative kinase 1 (PINK1)/parkin axis (Liu et al., 2020), signal transducer and activator of transcription 3 (STAT3) (Sun et al., 2021), and hypoxia inducible factor (HIF)-2α (Li et al., 2021). SIRT1 is widely expressed in normal renal tubular cells and podocytes, but decreases with renal diseases or ageing (Lim et al., 2012). Decreased SIRT1 activity leads to reduced production of peroxisome proliferator activated receptor γ coactivator-1a (PGC-1a) and autophagy (Lim et al., 2012), loss of resistance to ROS, suppression of FOXO, inhibition of AMPK, and activation of p53, resulting in cellular senescence and renal injuries (Goligorsky, 2020). Podocyte-specific reduction of SIRT1 promotes glomerulosclerosis and podocyte loss in mice (Chuang et al., 2017). Besides, capillary rarefaction is also related to the lack of SIRT1 in renal endothelial cells (Kida et al., 2016).

#### 4.2.6 Abnormality of immune system

For children with CKD, they show accelerated immune maturation and impaired immune function, and are forced into a state of premature immune senescence (George et al., 2017). Their CD4/CD8 ratio seems to be inverted, and CD57, a marker of senescence, is significantly increased, indicating the existence of immune senescence (George et al., 2017). Under the continuous stimulation of chronic inflammation in CKD, the replicative ability of T cells is impaired, leading to replicative senescence (Hayflick, 1965).

## 5 Targeting renal senescence in therapy of CKD

At present, the treatments of CKD are mainly focused on the etiology, symptoms and complications. When CKD progresses to end-stage renal disease (ESRD), renal replacement therapy, such as hemodialysis and peritoneal dialysis, is needed. However, the mortality of CKD patients is still high, and their life quality is low. Since senescence plays an important role in CKD, it could be assumed as a new target for CKD treatment (Tan et al., 2022). The approach of targeting senescence is known as senotherapy, which mainly includes senolytics, senomorphics, and rejuvenating agents (Goligorsky, 2020). The common agents are listed in Table 2. In recent years, numerous studies have confirmed their great potentials in ameliorating CKD and its complications (Knoppert et al., 2019; Wang et al., 2021).

**TABLE 2:** Therapeutic approaches against cellular senescence.

Senotherapy	Agents
Senolytics	dasatinib and quercetin Hickson et al., (2019), ABT-263 Chang et al., (2016), FOXO4-DRI Zhang et al., (2020), ABT-737 Lisowska et al., (2012), fisetin Zhu et al., (2017), 17-DMAG Litjens et al., (2006), A1331852 Zhu et al., (2017), A1155463 Zhu et al., (2017), panobinostat Samaraweera et al., (2017), BPTES Johmura et al., (2021), EGCG Kumar et al., (2019)
Senomorphics	metformin Kim et al., (2021), rapamycin Shavlakadze et al., (2018), bardoxolone Nagasu et al., (2019), pyrrolidine dithiocarbamate Okabe et al., (2013), methionine Wang et al., (2019), mitoq Xiao et al., (2017), SkQ1 Anisimov et al., (2011), ruxolitinib Griveau et al., (2020), flavonoids Lim et al., (2015)
Rejuvenating agents	resveratrol He et al., (2016), SRT1460 Zhao and Yu., (2021), SRT1720 Ren et al., (2017), SRT2183 He et al., (2010), D-Pinitol Koh et al., (2018), Isoliquiritigenin Huang et al., (2020), Rutin Khajevand-Khazaei et al., (2018), klotho Zou et al., (2018), PPAR-γ agonists Xu et al., (2020)
Others	calorie restriction Wang et al., (2021), exercise Kim et al., (2020), TA-65 Salvador et al., (2016), ACEI/ARB Jacobi et al., (2011), DHA Forman et al., (2020), immunomodulation Schroth et al., (2020)

ACEI, angiotensin-converting enzyme inhibitor; ARB, angiotensin receptor blocker; DHA, dehydroascorbic acid; EGCG, epigallocatechin gallate; FOXO4-DRI, Forkhead box O-4-D-Retro-Inverso; PPAR-γ, peroxisome proliferator-activated receptor-γ.

### 5.1 Nonpharmacologic approaches

CR mitigates senescence-associated renal changes by activating SIRT1 and AMPK, blocking mTOR and NF-κB signaling pathways, and inhibiting the activity of endothelin-1 (ET-1) (Wang et al., 2021), thereby promoting autophagy and reducing oxidative stress (Ning et al., 2013).

### 5.2 Senolytics

Senolytics eliminate senescent cells by promoting the pro-apoptotic pathways, inhibiting the SCAPs or activating the immune system (Sturmlechner et al., 2017). Preclinical studies have shown the exciting potential of multiple senolytics in reversing renal senescence (Knoppert et al., 2019). Dasatinib is a tyrosine kinase inhibitor that disturbs EFNB-dependent suppression of apoptosis, and quercetin is a natural flavonol that restrains PI3K and serpins (Wang et al., 2021). These two agents are often used in combination, and referred as “D + Q” (Wang et al., 2021). They are reported to alleviate senescence-related dysfunction in cell cultures and animal models (Xu et al., 2018). They can also significantly decrease the levels of p16, p21, SA-β-gal and SASPs in the adipose and skin tissues of patients with diabetic kidney diseases (Hickson et al., 2019). As the first generation senolytic in mice, ABT-263 inhibits the BCL-2 family, resulting in extensive apoptosis of senescent cells (Chang et al., 2016). FOXO4-DRI induces selective apoptosis of senescent cells by competitively inhibiting the FOXO4-p53 interaction, thus protecting renal function in aged mice (Zhang et al., 2020).

### 5.3 Senomorphics

Senomorphics are a group of SASP regulators, which can alleviate renal senescence in CKD (Schroth et al., 2020) by modulating a variety of pathways, such as MAPK, mTOR, NF-κB and NRF2 pathways (Iwasa et al., 2003). Metformin, an AMPK activator, has been proved to inhibit the induction of p16, p21, and SASPs, improve the function of mitochondrial complex I, activate autophagy (Piskovatska et al., 2019), reduce the production of ROS in cultured podocytes and prevent diabetes-induced renal hypertrophy (Lee et al., 2007; Piwkowska et al., 2010). A recent study showed that metformin exerts its anti-senescence effect by targeting senescent mesenchymal stem cells (MSC) in CKD (Kim et al., 2021). Since the side effects of metformin are minimal and are likely to be reversible, it is expected to be applicated in healthy individuals to block senescence-related renal changes (Barzilai et al., 2016). At present, the mTOR inhibitors mainly include rapamycin and its analog rapalog. They have attracted high attention in the treatment of renal diseases for their positive effect on renal senescence and fibrosis (Shavlakadze et al., 2018). However, their side effects are also significant, such as immunosuppression, infection and metabolic disorders (Fang et al., 2020). Therefore, they are not the best choice for healthy people to prevent renal senescence. Administration of pyrrolidine dithiocarbamate, an NF-κB inhibitor, alleviates renal interstitial fibrosis in rats (Okabe et al., 2013). Besides, inhibiting the activation of NF-κB at 24 h after AKI improves recovery of renal function and attenuates renal fibrosis (Johnson et al., 2017). The NRF2 agonist bardoxolone showed promising efficacy in CKD patients, but it was later discontinued because of the high rate of heart failure in patients randomly treated with it (de Zeeuw et al., 2013). Additionally, the potential oncogenic risks of NRF2 activators also need attention (Vega et al., 2018).

### 5.4 Rejuvenating agents

As an example of rejuvenating agent, resveratrol can improve senescence-related renal injury by activating SIRT1, reducing oxidative stress and inhibiting the pro-inflammatory SASPs (Wang et al., 2017). Besides, a variety of SIRT1 activators have been used to prevent and treat senesence-related renal deficiency (Han et al., 2021), such as SRT1460 (Zhao and Yu., 2021), SRT 1720 (Ren et al., 2017), SRT2183 (He et al., 2010), D-Pinitol (Koh et al., 2018), Isoliquiritigenin (Huang et al., 2020), and Rutin (Khajevand-Khazaei et al., 2018). Klotho expression can be stimulated by reactivation of endogenous klotho or supplement of exogenous klotho, so as to improve renal fibrosis and reduce senescence (Zou et al., 2018). Demethylation of the klotho gene promoter, klotho gene delivery and inhibition of histone deacetylase are potential strategies for the up-regulation of klotho (Zou et al., 2018). Several drugs have been reported to increase endogenous klotho (Zou et al., 2018), such as intermedin, and further alleviate senescence-related renal changes. In addition, direct administration of exogenous soluble klotho is also effective in improving the level of circulating klotho and preventing CKD.

### 5.5 Immunomodulation

Immunomodulatory therapy for senescence may be achieved by enhancing the tolerance to acute injury, inhibiting the pro-inflammatory state of senescent immune cells, and promoting the clearance of senescent cells. Peripheral tolerance is mainly controlled by dendritic cells (DCs) by inducing Tregs and T cell anergy. In a IRI model, the treatment of adenosine 2A receptor agonist *in vitro* can induce tolerogenic DCs, which further inhibit the activation of natural killer T (NKT) cells, thereby protecting the kidney (Li et al., 2012). Suppressing p38/MAPK in senescent CD8^+^ T cells improves their telomerase activity and mitochondrial function (Henson et al., 2014). Immunotherapies may also be used to eliminate senescent cells, such as reinfusion of *ex vivo* derived DCs, vaccines, and chimeric antigen receptor (CAR) T cells (Qudrat et al., 2017). By blocking the interaction between the non-classical major histocompatibility complex (MHC) molecule human leukocyte antigen-E (HLA-E) and the inhibitory receptor NKG2A expressed by NK and highly differentiated CD8^+^ T cells, the immune clearance of senescent cells can be improved (Pereira et al., 2019).

## 6 Future prospects

**FIGURE 4:**
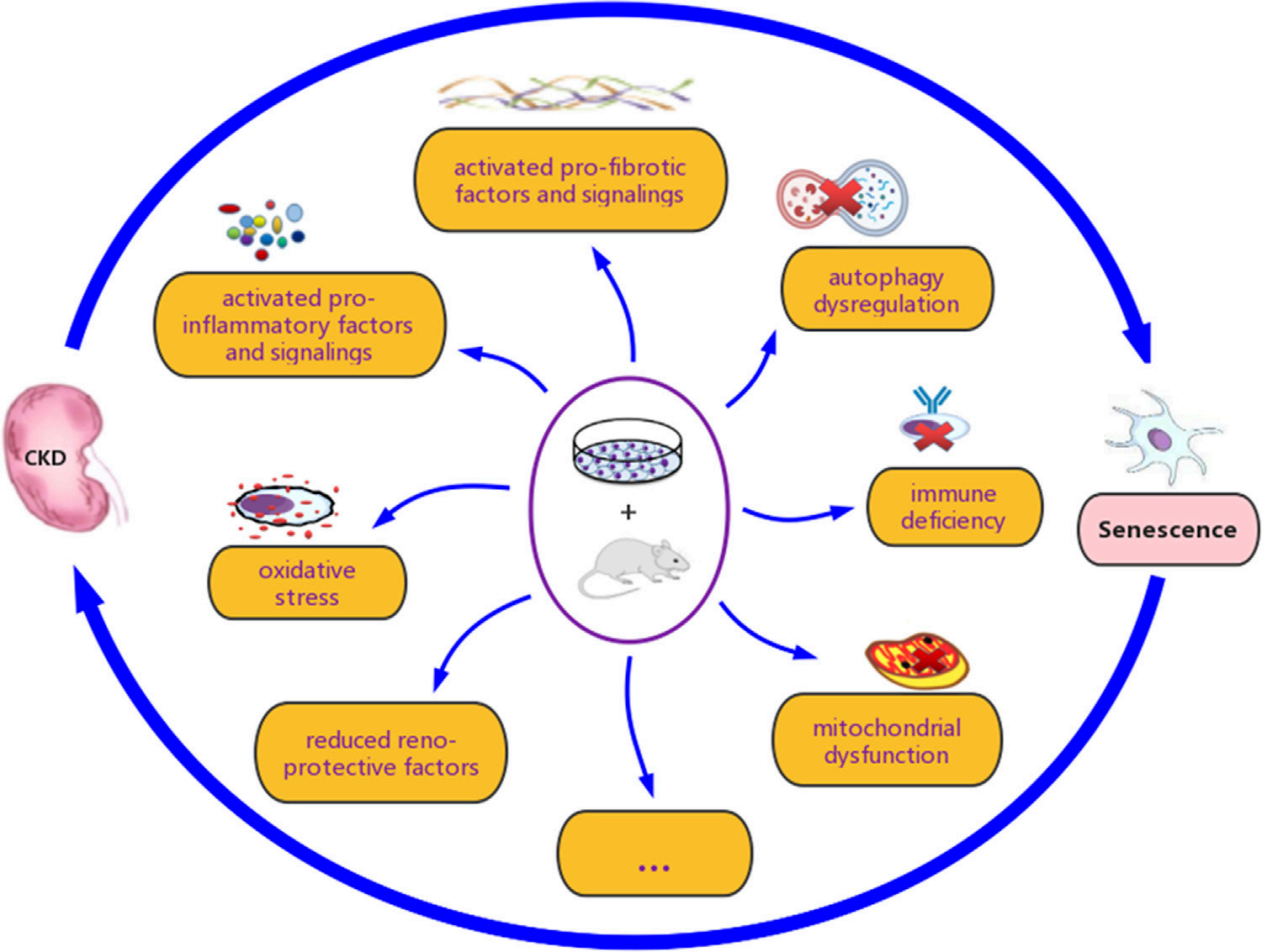
The interactive association between renal senescence and chronic kidney disease (CKD). Various studies have demonstrated that renal senescence and CKD are closely related, both *in vitro* and *in vivo*. Oxidative stress, mitochondrial dysfunction, loss of reno-protective factors, secretion of pro-inflammatory and pro-fibrotic factors, activation of associated signaling pathways, autophagy inhibition, and immune deficiency, are considered to be involved in their complex interaction.

Renal senescence and CKD share common characteristics and mechanisms, and there is a complex interactive relationship between them (Figure 4). Renal senescence is a promising target for therapeutic intervention of CKD, as preclinical data have shown the efficacy of senotherapies (Tan et al., 2022). In the future, more effective senotherapies and their judicious implementation are expected to fight against the progression of CKD or even reverse CKD, however, several challenges remained.

### 6.1 A deeper understanding of the pleiotropic effects of senotherapy

How to avoid the influence of senotherapy on the beneficial biological function of senescence and its potential toxicity to non-senescent cells and the whole organism? How to optimize dosing and limit adverse effects? These are remained major challenges. Therefore, it is necessary to conduct extensive researches on the pleiotropic effects of senotherapy.

### 6.2 Combing senotherapy with immunomodulation

On the one hand, senotherapy may enhance the dysregulated immune function in CKD, but immune-mediated CKD may deteriorate due to excessive immune activation (Schroth et al., 2020). On the other hand, the down-regulation of SASP caused by senotherapy will not only reduce cellular senescence, but also lead to the failure of recognizing SASP-inhibited senescent cells by immune cells and the reduction of SASP mediators involved in immune cell recruitment (Schroth et al., 2020), which will hinder immune-mediated clearance and further lead to the excessive accumulation of senescent cells. Therefore, it may be necessary to combine immunomodulation with senotherapy to achieve the triple therapy of specifically eliminating senescent cells, blocking their SASPs signaling, and promoting their immune targeting for CKD intervention (Schroth et al., 2020).

### 6.3 Heterogeneity identification of cellular senescence

Whether SASPs and SCAPs have specificity in different types of senescent cells? How to distinguish the short-term and long-term effects of senescent cells? Is there any difference between the response of primary senescence and secondary senescence to the current senotherapy? All these issues need further study. Another important point is the need for cell type-specific or tissue-specific identification of senescent cell markers. Single cell RNA sequencing can characterize and identify senescence on a single cell basis, which may help us to understand the dynamics and heterogeneity of senescent cells in affected organs. Targeted drug delivery to the kidney may further enhance the therapeutic effects of senotherapy on renal diseases and reduce its potential off-target effects.

### 6.4 Determination of the burden of senescent cells

The burden of senescent cells in the kidney may be a useful index for predicting renal prognosis (Liu et al., 2012). The problem is how to determine it. None of the current senescent markers are specific and unique. Besides, not all of the cells with these markers show senescent pathologies (Kirkland and Tchkonia, 2020), and different senescent subtypes are displayed when cells respond to the same stimulus (Chen et al., 2020). Limited by the detection methods, especially *in vivo*, the actual burden of senescent cells in CKD is still unclear. Additionally, whether the increase of senescent cells in renal biopsy can better predict CKD progression than existing markers requires prospective studies. It seems urgent to identify unique markers and convenient methods to detect and quantify senescence.
